# Supplementary material for: Exploring for-profit healthcare providers’ perceptions of inclusion in the Zambia National Health Insurance Scheme: A qualitative content analysis
Source: PLoS One. 2022 May 27;17(5):e0268940. doi: 10.1371/journal.pone.0268940 (PMC9140276; doi:10.1371/journal.pone.0268940)
Supplement: S1 Appendix — (DOCX) [file pone.0268940.s001.docx]

**Appendix 1. Interview guide**

**IN-DEPTH INTERVIEW GUIDE**

**For-Profit Private Health Care Providers in Zambia**

**Study Title:** Exploring for-profit healthcare providers’ perceptions of inclusion in the Zambia National Health Insurance Scheme: A qualitative content analysis

**Internal Review Board No:** 00005948

| Session ID | ____ \| ____ \| ____ |
| --- | --- |
| Date (DD/MM/YY): | ____ /____ / ____ |
| Interviewer (Name): | ______________________________________________ |
| Sign: | _______________________ |
| Provider/Facility (Name): | ______________________________________________ |
| Representative (Position): | _______________________ |
| Place/Region | District: _______________________  City:  Lusaka  Ndola  Kitwe |

1. **INTRODUCTION, INFORMATION AND CONSENT**
   1. Introduce yourself
   2. Statement of appreciation to participant
   3. Introduce the study and give purpose of the interview
   4. Information on participation (interview topics, session format, duration, voluntariness terms, risks involved, benefits for participation, confidentiality terms, consent and correspondence)
   5. Obtain written informed consent [READ WRITTEN CONSENT FORM]
2. Participant declines to participate?  Yes→ Stop the Interview
3. Consent dated and signed?  Yes

 No→Why____________________

1. Copy of consent given to participant?  Yes

 No→Why____________________

1. Any concern from participant?

________________________________________________________________

- - - 1. **Obtain permission to tape-record**

1. I would like to tape-record the interview so that I can remember everything we discuss here today. Is it okay with you if I tape-record?

 Yes → Turn on Recorder  No → Take notes

- - - 1. **Turn on a tape recorder and say session ID**

1. **INTERVIEW THEMES & QUESTIONS**
   1. **Awareness of NHI Scheme & Role in the Public Sector**
2. How well do you know the NHI Scheme?

Probes: Have you been talked to by any representative from NHIMA to explain the operations of the scheme? Have you read any documents on the NHI Scheme?

*NB: Provide a brief description of the NHI Scheme and NHIMA’s plan to include private providers in the scheme.*

*Focus: general knowledge about the NHI Scheme and its practices*

1. Do you see your facility as supplementary or complementary to the public sector in terms of health benefits package, clients, and catchment population?

Probes: supplementation/complementation in terms of health benefits package, geography (location), clients served and catchment population.

*NB: describe the terms supplementation/complementation if not understood.*

1. Do you have any partnership or external relations with the MoH or public health facilities? What is your role in these relations? Are there some gaps that you have identified between your facility and those in the public sector?

Probes: What are some of these gaps? What are some positive aspects of these gaps? What are their negative aspects?

- - 1. **Accreditation**

1. As a health care provider, are you considering applying for NHI accreditation?

Probes: If yes, would you need any assistance or information and what kind?

If no, why? What would it take for you to consider applying for accreditation?

*Focus: information on the accreditation process, requirements for accreditation, NHIMA’s health benefits package; contractual agreements; PPMs, and anything important from the informant’s perspective.*

1. What opportunities would the participation of your facility in the NHI Scheme create?

Probes: What opportunities would that create for your facility? What opportunities would that create for the public sector (especially for NHI accredited members)?

Service distribution; service efficiency, etc.

*Focus: Expectations, role in the public sector*

1. What do you see as other potential challenges (if any) for being accredited into the NHI Scheme?

Probes: Volume of delivered services/products; challenges with clients; challenges with NHIMA (requirements, reimbursements of claims, arrangement negotiations)

1. You are accredited and certified/licensed by the HPCZ and/or ZAMRA (Affirm).

How would you describe these processes in terms of easiness and difficulty of the requirements needed and steps are taken? Based on this experience, what would be your expectations about being accredited by NHIMA?

Probes: Some positive and negative aspects in the experience with accreditation; any impediments?

1. The fee structure for accrediting hospitals into the NHI for hospitals is K40 000 and as a clinic, lab, or pharmacy is K20 000 by NHIMA. Some may say this is expensive while others may say that it is cheap. What are your thoughts on these prescribed fees?
2. NHIMA will process applications of facilities that wish to be accredited within 90 days (3 months). What are your thoughts on this duration?
3. Is there anything else you would like to tell me on accreditation that we haven’t covered?
   - 1. **The Insurance Schemes**
4. Do you have any contractual agreements with any private insurance company?

 Yes ↆ Go to 12 and below

 No. Why have you not engaged in any contract with insurance companies?

Ask questions 15 and 16-20 hypothetically

1. Are any of your clients enrolled in some private health insurance scheme?

Probe: what is the estimate out of 10? What are your thoughts if these clients were members of the NHIS?

1. Why did you engage in partnerships with private health insurance?

Probes: what benefits and opportunities has this created for your facility and your clients?

1. What challenges do you frequently face (if any) with insurance companies? How do you resolve these challenges when they arise?

Probe: Payments for claims, duration of reimbursements, clarification of claims, etc.).

1. What are some major PPMs you have agreed to with your insurance companies? How do these PPMs square in light of NHIMA’s prescribed PPMs?

Probes: What are your thoughts concerning these PPMs in terms of satisfaction levels and their impacts on your facility finances?

1. What are major conditions that you as a healthcare provider has put forth for the insurance companies to meet in their partnerships with you? What conditions would you want to be satisfied in your accreditation into the NHIS?
2. How long does it take for your claims to be paid? Is this a reasonable reimbursement period? What happens if you are not reimbursed in time? How do you cover the expenses while you wait for your claims to be paid?
3. If NHIMA were to reimburse its clients within 90 days. Some may say this is too long whilst others think it’s too short. What are your thoughts on this?
4. The membership card provided for users by NHIMA will have a picture, and details of a member with a biometric code at the back. One may rate this as a good security measure, while others not. What are your thoughts on this? How does this compare to cards from other health insurance companies?
5. Some private healthcare providers may prefer operations such as processing claims and reimbursements to be managed manually or while others electronically. How would you compare these in terms of efficiency and security management both to you as a provider and to the insurance company?
6. Is there anything else you would like to tell me about your experience with private insurance companies that I have not covered so far?
7. **DEMOGRAPHIC INFORMATION**

| Interview Session ID | ____ \| ____ \| ____ (enter as given by interviewer) |
| --- | --- |
| Date (DD/MM/YY): | ____ /____ / ____ |

| **1. Gender** | 󠇟 Male 󠇟 Female |
| --- | --- |
| **2. Age (yrs.)** | 1. 󠇟 ≤ 30 󠇟󠅼31-40 󠇟󠅼 41-50 󠇟󠇟 51-60 󠇟󠇟 61-70 󠇟󠇟 71 ≤ 󠇟 |
| **3. Education Qualification** |  PhD /Master's Degree 󠅼󠅼 Medical/Bachelor’s Degree 󠅼󠇟 Diploma󠇟  󠅼 College/Trade/Vocational Certificate 󠅼󠅼 Secondary School Certificate |
| **4. Technical Qualification** |  Medical doctor  Dentist 󠅼󠅼 Anaesthesiologist 󠅼 󠅼󠇟 Clinical officer  󠇟 Nurse 󠅼󠅼 Midwife 󠅼󠅼 Medical assistant 󠅼󠇟 Auxiliary nurse 󠇟 󠅼󠇟 Health assistant 󠅼 󠅼󠇟 Radiologist 󠅼 󠅼󠇟 Lab technologist 󠅼 󠅼 󠅼󠇟 Pharmacist 󠇟 󠅼󠇟 Public health worker  󠇟 Others _____________________________ (please specify) |
| **5. Years of Experience** | 1. 󠇟1-2 󠇟 󠅼󠅼 3-4 󠇟 5-6 󠇟7-8 󠇟9-10 󠇟11≤ |
| **8. Owner of Facility?** | 󠅼 Yes  󠅼 No _____________________________ (please specify position title) |
| **7. Years of Operation** | 󠇟1-4 󠇟 󠅼󠅼 5-8 󠇟 9-12 󠇟13-16 󠇟17-20 󠇟21≤ |
| **8. Number of medical staff** | __________ |
| **9. Accreditation** |  HPCZ  ZAMRA |
| **10.** When did you become accredited or registered? Month ____ Year_______ | |

**INTERVIEW GUIDE THEMES**

**Experiences (with the Public, Govt., HPCZ/ZAMRA, Insurance / PICs) Participating in the NHIS**
